# Trends in incidence and histological pattern of thyroid cancer in Ho Chi Minh City, Vietnam (1996–2015): a population-based study

**DOI:** 10.1186/s12885-021-08023-z

**Published:** 2021-03-20

**Authors:** Dung X. Pham, Hien D. Nguyen, An H. T. Phung, Tung D. Bui, Thach S. Tran, Bich N. H. Tran, Lan T. Ho-Pham, Tuan V. Nguyen

**Affiliations:** 1 Ho Chi Minh City Oncology Hospital, Ho Chi Minh City, Vietnam; 2 Department of Oncology, Pham Ngoc Thach University of Medicine, Ho Chi Minh City, Vietnam; 3 BioMedical Research Center, Pham Ngoc Thach University of Medicine, Ho Chi Minh City, Vietnam; 4 Bone and Muscle Research Group, Ton Duc Thang University, Ho Chi Minh City, Vietnam; 5 Department of Healthcare Directions, Ho Chi Minh City Oncology Hospital, Ho Chi Minh City, Vietnam; 6 Bone Biology Division, Garvan Institute of Medical Research, Sydney, Australia; 7 Bureau of Health Information, St Leonards, Sydney, New South Wales Australia; 8 St Vincent’s Clinical School, UNSW Medicine, UNSW Sydney, Sydney, Australia; 9 School of Biomedical Engineering, University of Technology Sydney, Sydney, Australia

**Keywords:** Thyroid cancer, Incidence, Histological pattern, Trends, Vietnam

## Abstract

**Background:**

The burden and trend of thyroid cancer in Vietnam have not been well documented. This study aimed to investigate the trends in incidence and histological pattern of thyroid cancer in Ho Chi Minh City from 1996 to 2015.

**Methods:**

A population-based study retrieved data from the Ho Chi Minh City Cancer Registry during 1996–2015. Trends in the incidence of thyroid cancer were investigated based on age, gender, and histology for each 5-year period. Annual percentage change (APC) in incidence rates was estimated using Joinpoint regression analysis.

**Results:**

In the study period, there were 5953 thyroid cancer cases (men-to-women ratio 1:4.5) newly diagnosed in Ho Chi Minh City with the mean age of 42.9 years (±14.9 years). The age-standardized incidence rate of thyroid cancer increased from 2.4 per 100,000 during 1996–2000 (95% confidence interval [95% CI]: 2.2–2.6) to 7.5 per 100,000 during 2011–2015 (95% CI: 7.3–7.9), corresponded to an overall APC of 8.7 (95% CI 7.6–9.9). The APC in men and women was 6.2 (95% CI: 4.2–8.2) and 9.2 (95% CI: 8.0–10.4), respectively. The incidence rate in the < 45 years age group was the highest diagnosed overall and increased significantly in both men (APC 11.0) and women (APC 10.1). Both genders shared similar distribution of subtype incidences, with papillary thyroid cancer constituted the most diagnosed (73.3% in men and 85.2% in women). The papillary thyroid cancer observed a markedly increase overall (APC of 10.7 (95% CI 9.3–12.0)).

**Conclusions:**

There were appreciable increases in the age-standardized incidence rate of thyroid cancer in both genders, mainly contributed by the papillary subtype. The age of patients at diagnosis decreased gradually. The widespread utilization of advanced diagnostic techniques and healthcare accessibility improvement might play a potential role in these trends. Further investigations are needed to comprehend the risk factors and trends fully.

## Background

Thyroid cancer is the most widespread neoplasm of the endocrine system. The data of the American National Cancer Institute indicated that thyroid cancer incidence increased at a 6.2% rate per year between 1997 and 2006, emerging as one of the fastest increasing cancer in both genders [1]. In the United States, the increasing incidence of thyroid cancer over a period from 1973 to 2002 proved to be predominantly due to advanced detection, particularly with the advent of ultrasound and fine-needle aspiration [2].

Women experience a higher incidence of thyroid cancer than their men counterparts, with the women-to-men ratio is consistently high (approximately 3:1), especially after puberty and during the reproductive years [3]. In women, the peak occurrence of thyroid cancer is during their reproductive period, and at the time of menopause and postmenopause, incidence rates exhibit a decline. Patterns relating to hormonal and reproductive factors may be partly attributable to this gender-specific trend and unusual age distribution in this type of cancer. Although the median age at diagnosis for most cancer types is over 60 years, it is only at around age 48 for thyroid cancer [4]. Other risk factors of thyroid neoplasm are a history of benign thyroid diseases (adenomas, nodules, goiters), a high iodine intake, radiation, and long-term smoking or alcohol [3].

As regards tumor histology, there are five different types of thyroid cancer: papillary thyroid carcinoma, follicular thyroid carcinoma, medullary thyroid carcinoma, anaplastic thyroid carcinoma, and others. About 95% of thyroid cancers originate from the follicular epithelium, and they are papillary, follicular, and anaplastic forms. The most common type is papillary, which has a proportion of 70% or more in many countries surveyed [3]. The frequency of follicular carcinoma ranges from about 10 to 40%, basing on the region’s iodine status. Because it may be difficult to distinguish between benign and malignant follicular neoplasms, leading to some under- or over-reporting of follicular thyroid cancer. Anaplastic and medullary carcinomas account for about 5% of total thyroid cancer cases. The prognosis is extremely good for papillary and follicular thyroid cancer, while the anaplastic carcinoma prognosis proved relatively aggressive [3].

In Vietnam – one of the Southeast Asian nations with a population of 97 million, there are still no papers investigating thyroid cancer burden and trends of age, gender, and tumor histology. In this paper, we aim to analyze the trends in incidence and histological patterns of thyroid cancer in Ho Chi Minh City from 1996 to 2015, providing a regional perspective to monitor and comprehend the changing and spur additional research into the causes of these observed patterns.

## Methods

### Data source

A population-based study was conducted using data extracted from the Ho Chi Minh City Cancer Registry from 1996 to 2015. We performed this study under the approval of the Ethics Committee of the Oncology Hospital of Ho Chi Minh City. The need of the written informed consent has waived by the Ethics Committee of the Oncology Hospital of Ho Chi Minh City.

Registry data were collected and assessed, adhering to guidelines from the International Agency for Research on Cancer and the International Association of Cancer Registries for low and middle-income countries. The Ho Chi Minh City Cancer Registry used the ICD-10 code for thyroid cancer diagnosis (C73). The histology types of thyroid cancer were categorized into five subtypes according to the International Classification of Diseases for Oncology, third edition (ICD-O-3) as papillary carcinoma (ICD-O-3 codes 8050, 8260, 8340, 8341, 8343, 8344, and 8350), follicular carcinoma (8290, 8330–8332, and 8335), medullary carcinoma (8345, 8346, and 8510), anaplastic carcinoma (8012, 8020, 8021, and 8030–8032), and other or unspecified thyroid cancer (all other ICD-O-3 codes) [5].

Population statistics per gender and age during 1996–2015 were obtained from census data managed by the General Statistics Office (GSO) of Ho Chi Minh City and the Bureau of Statistics of Vietnam.

### Statistical analysis

From the population statistics of Ho Chi Minh City, we computed the thyroid cancer incidence rate for four consecutive periods: 1996–2000, 2001–2005, 2006–2010, and 2011–2015. The data was aggregated into every 5 years because of some low number in incidences, which would enhance the statistical computing stability.

The age-standardized incidence rate was calculated as the number of newly diagnosed people with thyroid cancer per 100,000 individuals by gender and histology subtype, using Segi’s world population. Age group categories were selected as < 45, 45–64, and + 65 years.

Joinpoint regression analysis was estimated by Joinpoint regression software version 4.8 to identify statistically significant trend change points across consecutive periods [6]. Joinpoint tests of significance use a Monte Carlo permutation method, and annual percentage changes (APCs) were estimated utilizing generalized linear models assuming a Poisson distribution [7]. Data were reported as mean (standard deviation, SD) for continuous variables, and frequency and percentage for categorical variables. Comparisons between two groups were done using an unpaired Student’s t-test for continuous data and Chi-square test for categorical data. All hypothesis tests were two-sided. A *p*-value < 0.05 was considered to be statistically significant.

## Results

During 1996–2015, there were 5953 thyroid cancer cases (1078 men and 4875 women) newly diagnosed in Ho Chi Minh City with the mean age of 42.9 years (±14.9 years) (Table 1). Overall, the age at diagnosis decreased gradually over time, from a mean age of 46.3 years (±18.3 years) in 1996–2000 to 42.2 years (±13.8 years) in 2011–2015 (*p* < 0.001). Men patients were slightly older than women patients (mean age: 44.9 versus 42.5 years, respectively, *p* < 0.001). Stratified by age group, both genders share a relatively similar subgroups’ proportion. Thyroid cancer was diagnosed mostly in < 45 years patients, comprised of 50.7% in men and 58.1% in women. The 65+ years age group had the lowest percentage, which decreased consistently and markedly by 70% in both men and women throughout the period.

**Table 1:** Characteristics of thyroid cancer patients in Ho Chi Minh City, 1996–2015

	Total (*N* = 5953)	1996–2000 (*N* = 535)	2001–2005 (*N* = 861)	2006–2010 (*N* = 1732)	2011–2015 (*N* = 2825)	*p*-Value
**Gender**						
Men	1078 (18.1)	132 (24.7)	158 (18.4)	288 (16.6)	500 (17.7)	< 0.001
Women	4875 (81.9)	403 (75.3)	703 (81.6)	1444 (83.4)	2325 (82.3)	
Men-to-women ratio	1:4.5	1:3.0	1:4.5	1:5	1:4.5	
**Age at diagnosis**						
Total	42.9 ± 14.9	46.3 ± 18.3	43.7 ± 16.3	42.6 ± 14.8	42.2 ± 13.8	< 0.001
Men	44.9 ± 16.4	48.7 ± 18.9	48.6 ± 16.8	44.0 ± 16.8	43.2 ± 15.0	
Women	42.5 ± 14.6	45.5 ± 18.1	42.6 ± 16.0	42.3 ± 14.3	42.0 ± 13.5	
p-Value	< 0.001	0.079	< 0.001	0.099	0.110	
**Age group (years) Men**						< 0.001
< 45	547 (50.7)	54 (40.9)	62 (39.2)	146 (50.7)	285 (57)	
45–64	395 (36.6)	46 (34.8)	61 (38.6)	109 (37.8)	179 (35.8)	
65+	136 (12.6)	32 (24.2)	35 (22.2)	33 (11.5)	36 (7.2)	
**Age group (years) Women**						< 0.001
< 45	2831 (58.1)	221 (54.8)	409 (58.2)	837 (58.0)	1364 (58.7)	
45–64	1651 (33.9)	108 (26.8)	213 (30.3)	497 (34.4)	833 (35.8)	
65+	393 (8.1)	74 (18.4)	81 (11.5)	110 (7.6)	128 (5.5)	
**Histology Men**						
Papillary	790 (73.3)	72 (54.5)	103 (65.2)	223 (77.4)	392 (78.4)	
Follicular	17 (1.6)	8 (6.1)	4 (2.5)	2 (0.7)	3 (0.6)	
Medullary	16 (1.5)	5 (3.8)	3 (1.9)	2 (0.7)	6 (1.2)	
Anaplastic	15 (1.4)	3 (2.3)	6 (3.8)	0 (0.0)	6 (1.2)	
Other	240 (22.3)	44 (33.3)	42 (26.6)	61 (21.2)	93 (18.6)	
**Histology Women**						
Papillary	4154 (85.2)	269 (66.7)	570 (81.1)	1254 (86.8)	2061 (88.6)	
Follicular	77 (1.6)	34 (8.4)	19 (2.7)	13 (0.9)	11 (0.5)	
Medullary	13 (0.3)	4 (1.0)	4 (0.6)	2 (0.1)	3 (0.1)	
Anaplastic	26 (0.5)	4 (1.0)	5 (0.7)	9 (0.6)	8 (0.3)	
Other	605 (12.4)	92 (22.8)	105 (14.9)	166 (11.5)	242 (10.4)	

Numbers of each gender, age and histological group are presented as frequency (%) and ages are presented as mean years ± SD

Regarding histological patterns, both men and women had the subtype incidence distribution (Table 1). Papillary thyroid cancer constituted the most frequently diagnosed, with 790 (73.3%) cases in men and 4154 (85.2%) cases in women. The second highest diagnosis was the other and unspecified thyroid cancer, with 240 (22.3%) cases in men and 605 (12.4%) in women. The incidences of follicular, medullary, and anaplastic thyroid cancer were very low in both genders, contributed 96 (1.6%), 29 (0.5%), and 41 (0.7%) cases, respectively overall.

**Fig. 1:**
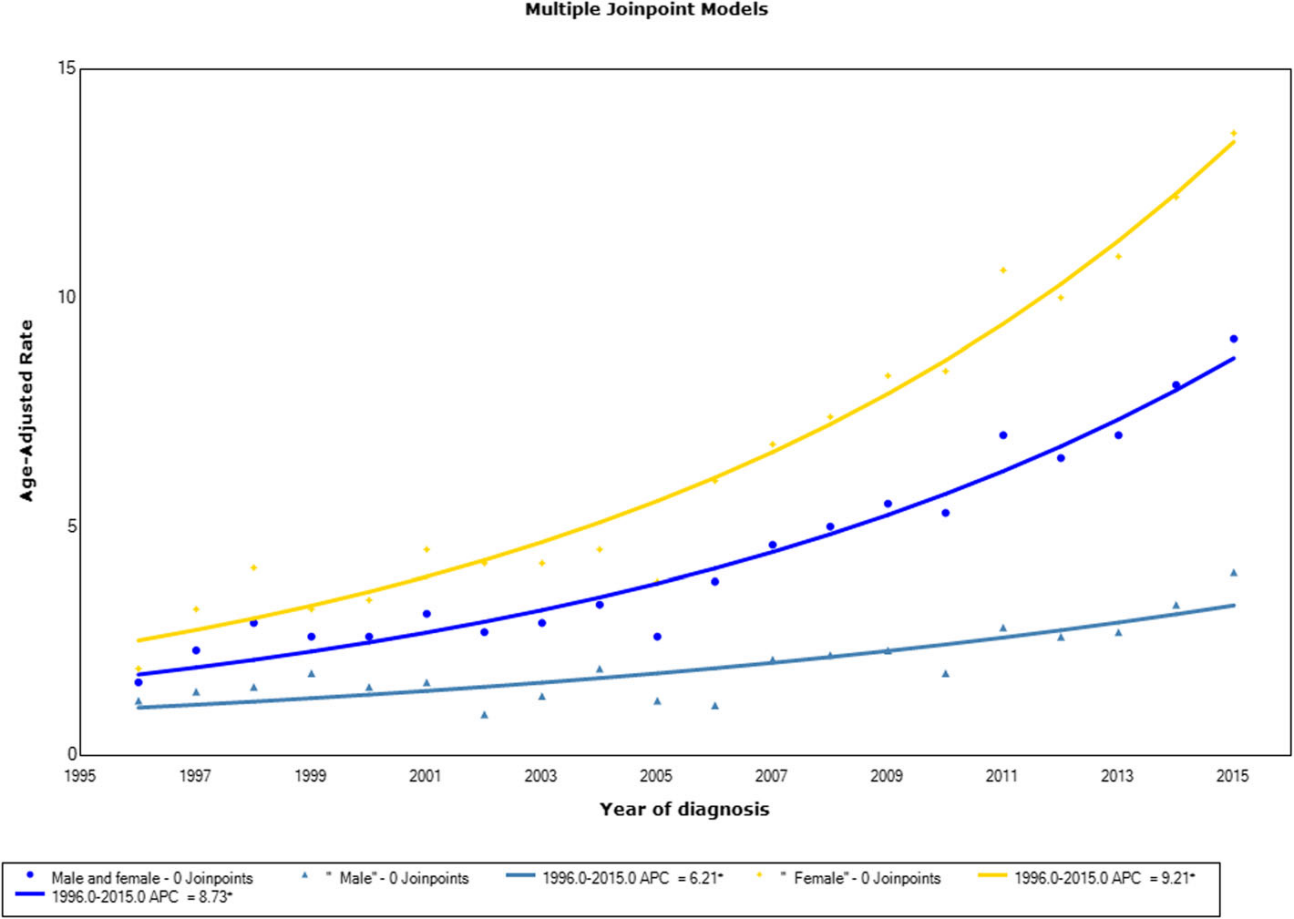
Joinpoint regression curve of age-standardized incidence rates for thyroid cancer by gender in Ho Chi Minh City, 1996–2015

The age-standardized incidence rate of thyroid cancer increased from 2.4 per 100,000 during 1996–2000 (95% confidence interval [95% CI]: 2.2–2.6) to 7.5 per 100,000 during 2011–2015 (95% CI: 7.3–7.9); a 3.1-fold increase (*p* < 0.05 for trend) (Table 2). This increase corresponded to an estimated APC of 8.7 (95% CI 7.6–9.9) (Fig. 1). Both genders observed the increasing trend in thyroid cancer incidence, and women had a greater increase in overall APC (9.2, 95% CI: 8.0–10.4) compared with men (6.2, 95% CI: 4.2–8.2).

**Table 2:** Incidence rate and annual percentage change of thyroid cancer by gender in Ho Chi Minh City, 1996–2015

	Estimate (95% CI)	1996–2000	2001–2005	2006–2010	2011–2015	Overall
**Overall**	Unadjusted	2.0 (1.9–2.2)	2.7 (2.5–2.9)	4.8 (4.6–5.0)	7.5 (7.2–7.8)	4.5 (4.4–4.6)
	Standardized^a^	2.4 (2.2–2.6)	2.9 (2.7–3.1)	4.8 (4.6–5.1)	7.5 (7.3–7.9)	4.7 (4.6–4.8)
	APC	9.1 (−7.9–29.3)	−1.5 (−11.8–9.9)	9.2 (1.8–17.1)	7.6 (−0.5–16.3)	8.7 (7.6–9.9)
**Men**	Unadjusted	1.0 (0.9–1.2)	1.0 (0.9–1.2)	1.7 (1.5–1.9)	2.8 (2.5–3.0)	1.7 (1.6–1.8)
	Standardized^a^	1.5 (1.2–1.8)	1.4 (1.1–1.6)	1.9 (1.7–2.2)	3.1 (2.8–3.4)	2.1 (1.9–2.2)
	APC	6.4 (−5.1–19.2)	0.8 (−26.5–38.2)	9.9 (−18.1–47.5)	10.2 (− 1.2–22.9)	6.2 (4.2–8.2)
**Women**	Unadjusted	3.0 (2.7–3.3)	4.3 (3.9–4.6)	7.7 (7.3–8.1)	11.9 (11.4–12.4)	7.1 (6.9–7.3)
	Standardized^a^	3.2 (2.9–3.5)	4.2 (3.9–4.6)	7.4 (7.0–7.8)	11.5 (11.0–12.0)	7.0 (6.8–7.2)
	APC	9.0 (−17.6–44.3)	−2.9 (−8.8–3.3)	9.0 (5.1–13.0)	7.3 (0.9–14.0)	9.2 (8.0–10.4)

^a^Standardization to Segi’s world population

**Fig. 2:**
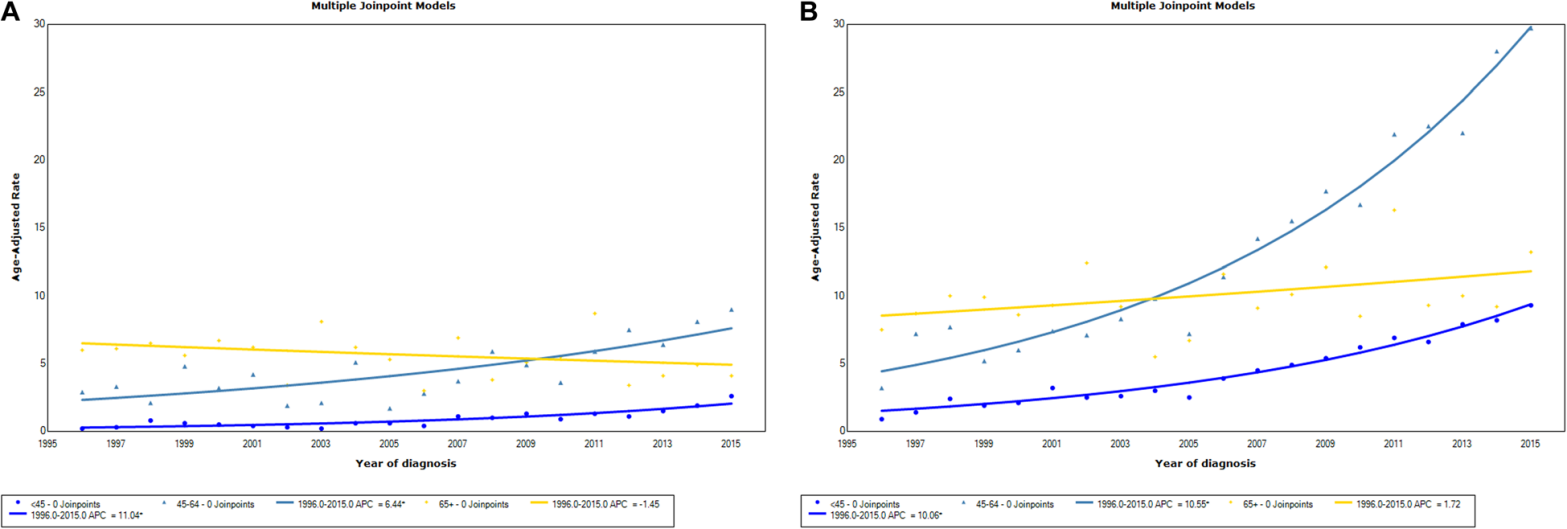
Joinpoint regression curve of age-standardized incidence rates for thyroid cancer by age group in Ho Chi Minh City, 1996–2015

Among men, there was a slight decrease in age-standardized incidence rate from 1.5 per 100,000 in 1996–2000 to 1.4 per 100,000 in 2001–2005, followed by a steady increase to 3.1 per 100,000 in 2011–2015 (a 2.1-fold increase overall). Whereas the incidence in < 45 years age group increased significantly with an estimated APC of 11.0 (95% CI: 8.3–13.9), + 65 years age group incidence decreased with an estimated APC of − 1.5 (95% CI: − 3.8-0.9) (Fig. 2a).

Among women, the age-standardized incidence rate increased consistently and substantially from 3.2 per 100,000 in 1996–2000 to 11.5 per 100,000 in 2011–2015 (a 3.6-fold increase overall). The rate increased in all age groups, with the highest in the 45–64-year-old patients (APC of 10.5 (95% CI: 9.2–11.9)) (Fig. 2b).

**Fig. 3:**
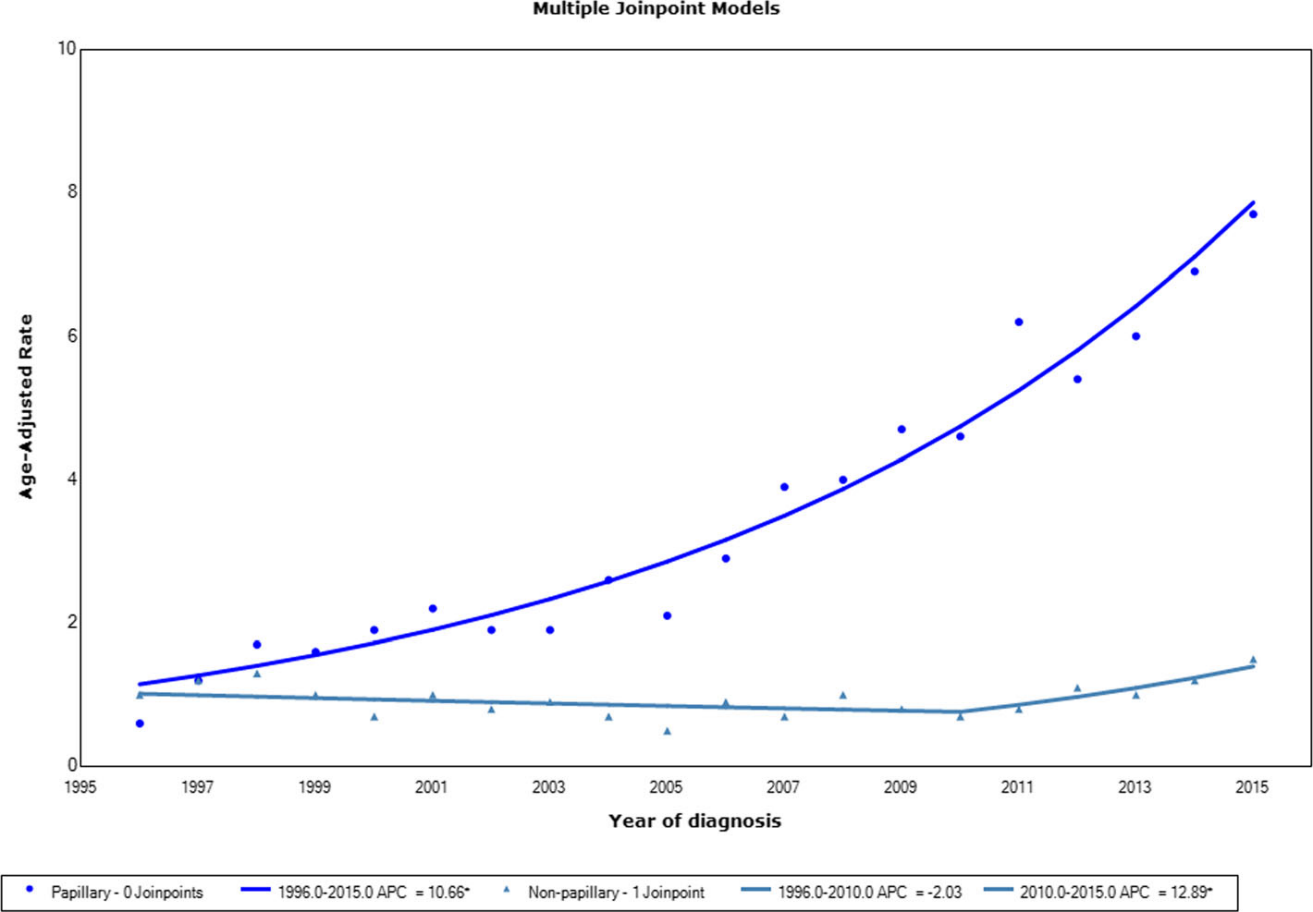
Joinpoint regression curve of age-standardized incidence rates for papillary and non–papillary thyroid cancer in Ho Chi Minh City, 1996–2015

During 1996–2015, the age-standardized incidence rate of papillary thyroid cancer increased consistently and markedly from 0.6 (95% CI 0.4–0.9) to 7.7 (95% CI 7.0–8.4), a 12.8-fold increase. The overall APC of papillary thyroid cancer was 10.7 (95% CI 9.3–12.0) (Fig. 3). Joinpoint analysis for non-papillary thyroid cancer trend detected one joinpoint in 2010 with an average APC of 1.7 (95% CI -1.6-5.1). The APC was − 2.0 (95% CI − 4.8-0.8) during 1996–2010 and 12.9 (95% CI 1.2–25.9) during 2010–2015.

## Discussion

Our study shows that the age-standardized incidence rate of thyroid cancer in Ho Chi Minh City increased appreciably in both genders during 1996–2015. The women experienced a greater incidence rate and APC than men. Regarding age at diagnosis, men were slightly older than women patients. The overall age at diagnosis appeared to decrease gradually, and the highest incidence was in the < 45 years age group. Both genders shared a similar distribution of histological subtypes incidences. Papillary thyroid cancer was the most frequently diagnosed subtype and had a marked increase of 12.8-fold in age-standardized incidence rate.

The appreciably increases in the age-standardized incidence rate of thyroid cancer in Ho Chi Minh City for both genders are in accord with patterns observed in other populations over the last few decades. In China (2005–2015), the APC of overall, men, and women were 12.4, 13.3, and 10.7, respectively [8]. In Sri Lanka (2001–2010), the APC of overall, men, and women were 8.2, 5.3, and 9.0, respectively [9]. In Taiwan (1997–2012), the APC of overall, men, and women were 10.0, 9.9, and 10.3, respectively [10]. The Ho Chi Minh City figures are comparable, with the same higher trend in women than men: the APC of overall, men, and women were 8.7, 6.2, and 9.2, respectively.

There are several reasons for this upward trend in thyroid cancer incidence. Firstly, the widespread utilization of advanced diagnostic techniques could explain this acceleration rate [11, 12]. The introduction of computed tomography (CT) scan, magnetic resonance imaging, high-resolution thyroid ultrasonography, and fine-needle aspiration significantly enhanced diagnostic sensitivity. Additionally, there has been an apparent increase in medical practitioners skilled in diagnosing thyroid neoplasms in Vietnam. Secondly, it is possible that increased levels of healthcare access contributed substantially to detection improvement. Thyroid cancer incidence was significantly positively correlated with the multidimensional measures of access to healthcare [13]. As with socioeconomic status development, Vietnamese people could get to healthcare services easier and more frequently. This accessibility might be the rationale why the age at diagnosis decreased gradually since the malignancy could be detected before manifesting obvious symptoms. Thirdly, the excessive exposure to radiation from the escalating use of CT scans might also increase the thyroid cancer incidence rate [14, 15]. During childhood, the neck CT examinations could increase the risk of developing thyroid neoplasms up to 390 per million exposed individuals [16]. Finally, there are some likely risk factors related to thyroid cancer incidence, such as obesity, red meat, processed food consumption, iodine intake, mental factors, and environmental pollutant [17–20]. These factors should be considered in further studies to clarify their roles in the observed increase of thyroid cancer in Vietnam.

The overall men-to-women ratio of 1:4.5 in our study was reasonably consistent with other studies [1, 8–10]. The polymorphism in estrogen receptors is supposed to be the molecular factor that results in this gender disparity [21]. Estrogen can substantially increase the cell proliferation rate in thyroid cancer cell lines, encouraging thyroid dysplasia and developing to malignancy [22, 23]. Moreover, it is hypothesized that fertility drugs are potential risk factors for thyroid cancer [24]. In agreement with various published data, women patients were slightly younger than men patients at the time of diagnosis [9, 25, 26]. This difference has yet to be explained, but the women’s hormones increase during pregnancy might play a potential role. Otherwise, menstrual and reproductive factors have a weak association with thyroid cancer [27–29].

According to histological results, papillary thyroid cancer was the main driver of the overall thyroid cancer increase during 1996–2015. This pattern reflected as well with previous findings worldwide [8–10, 30, 31]. It has been postulated that this escalating trend in thyroid cancer incidence was not a real increase but an overdiagnosis of papillary thyroid cancer, particularly with small and localized tumors due to widespread advanced diagnostic techniques [11, 32]. Papillary is the most common but the least aggressive subtype in thyroid cancer since it tends to progress very slow [33]. The 5-year relative survival rates in papillary thyroid cancer cases without metastases were nearly 100% [34]. Overdiagnosis of papillary thyroid cancer could put patients in the harm of overtreatment (surgical complications, iatrogenic hypothyroidism) without really improving the overall prognostic. Besides, overtreatment would increase the healthcare burden. Due to our study’s lack of data, we need further investigations, including diagnostic techniques, tumor characteristics, treatment procedures, and mortality, to confirm the overdiagnosis hypothesis for Ho Chi Minh City’s thyroid incidence trend.

Our study has some limitations. Firstly, there are incomplete data collections of diagnostic techniques, tumor characteristics (size, stage), treatment procedures, and mortality, limiting the full comprehension of the thyroid incidence trend. Secondly, the Ho Chi Minh Cancer Registry data did not include information on relevant characteristics, including socioeconomic status, menstrual and pregnancy factors, BMI, diet, environmental factors, and family history, preventing us from assessing specific discrepancies and risk factors. Nonetheless, the Ho Chi Minh Cancer Registry constitutes a comprehensive thyroid cancer database of all hospitals in Ho Chi Minh City for decades. Its coverage could represent of thyroid cancer database in the South of Vietnam.

## Conclusions

There were appreciable increases in the age-standardized incidence rate of thyroid cancer in both genders, mainly contributed by the papillary subtype. The age of patients at diagnosis decreased gradually. The widespread utilization of advanced diagnostic techniques and healthcare accessibility improvement might play a potential role in these trends. Further investigations, including socioeconomic status, menstrual and pregnancy factors, BMI, diet, environmental factors, family history diagnostic techniques, tumor characteristics, treatment procedures, and mortality are needed to fully comprehend the risk factors and trends.

## Data Availability

Data from all registries was collected and assessed based on guidelines from the International Agency for Research on Cancer and the International Association of Cancer Registries, adapted to a low and middle-income context. Registry data are stored in a computerized data at the Oncology Hospital of Ho Chi Minh City. The dataset includes the following variables: age, sex, diagnosis, year of diagnosis, cancer stage, treatment, and survival status. All authors had no special access privileges in accessing these datasets, which other interested researchers would not have. Age-and-gender population statistics were obtained from census data managed by the General Statistics Office (GSO) of Ho Chi Minh City. Age-and-gender population statistics in 1999 for Vietnam were obtained from the Bureau of Statistics of Vietnam. There is no URL for the population data. However, requests for data access can be made to the General Statistics Office of Ho Chi Minh City, contact through email tdnlinh@ump.edu.vn.

## Abbreviations

APC: Annual percentage change
BMI: Body mass index
CI: Confidence interval
CT: Computed tomography
GSO: General Statistics Office
ICD: The International Statistical Classification of Diseases and Related Health Problem
ICD-O: The International Classification of Diseases for Oncology
SD: Standard deviation
